# A Microfluidic Detection System for Bladder Cancer Tumor Cells

**DOI:** 10.3390/mi10120871

**Published:** 2019-12-11

**Authors:** Shuxing Lv, Jinwei Yu, Yan Zhao, Hongxiang Li, Fang Zheng, Ning Liu, Dahua Li, Xuguo Sun

**Affiliations:** 1 School of Medical Laboratory, Tianjin Medical University, Tianjin 300203, China; lyushuxing@gmail.com (S.L.); jinweiyu2008@163.com (J.Y.); zhengfang@tmu.edu.cn (F.Z.); 2 School of Computer Science and Engineering, Tianjin University of Technology, Tianjin 300384, China; zhaoyan0612@163.com (Y.Z.); xiangaler@163.com (H.L.); lidah2005@163.com (D.L.); 3 Pillar of Engineering Product Development, Singapore University of Technology and Design, Singapore 487372, Singapore; liun0006@e.ntu.edu.sg

**Keywords:** bladder cancer, tumor cell detection, microfluidics, cell image recognition, detection systems

## Abstract

The clinical characteristics of excreted tumor cells can be found in the urine of bladder cancer patients, meaning the identification of tumor cells in urine can assist in bladder cancer diagnosis. The presence of white blood cells and epithelial cells in the urine interferes with the recognition of tumor cells. In this paper, a technique for detecting cancer cells in urine based on microfluidics provides a novel approach to bladder cancer diagnosis. The bladder cancer cell line (T24) and MeT-5A were used as positive bladder tumor cells and non-tumor cells, respectively. The practicality of the tumor cell detection system based on microfluidic cell chip detection technology is discussed. The tumor cell (T24) concentration was around 1 × 10^4^ to 300 × 10^4^ cells/mL. When phosphate buffer saline (PBS) was the diluted solution, the tumor cell detected rate was 63–71% and the detection of tumor cell number stability (coefficient of variation, CV%) was 6.7–4.1%, while when urine was the diluted solution, the tumor cell detected rate was 64–72% and the detection of tumor cell number stability (CV%) was 6.3–3.9%. In addition, both PBS and urine are tumor cell dilution fluid solutions. The sample was analyzed at a speed of 750 microns per hour. Based on the above experiments, a system for detecting bladder cancer cells in urine by microfluidic analysis chip technology was reported. The rate of recognizing bladder cancer cells reached 68.4%, and the speed reached 2 mL/h.

## 1. Introduction

Bladder cancer is a common cancer of the urinary system and the ninth most common malignant tumor in the world [1]. The presence of bladder cancer cells in the urine provides important evidence for bladder cancer diagnosis [2]. One of the reasons for the low sensitivity of urine cytology is the interference of background cells and other impurities [3].

Urine examination, which has now been automated, constitutes one of the most common diagnostic tests in clinical laboratories, second only to serum chemistry and complete blood counts. The urine sediment analyzer can be divided into two different systems according to its functional principle: one is a digital-image-based system, and the other is a flow-cytometry-based system. The digital-image-based system analyzes urine deposits based on several images taken with a built-in digital camera and then performs automatic particle recognition and sorting [4,5]. Moreover, more detailed analyzers use digital-image-based systems, including most mainstream manufacturers, such as Roche, Siemens, and Beckman. [4,6]. However, this method still fails to completely solve the low sensitivity of urine cytology [7].

Studies have shown that microfluidic technology can be used in unspecialized laboratories to shorten and simplify previously cumbersome tasks [8]. Droplet-based microfluidic techniques have been widely used as efficacious tools for single-cell analysis [9]. Current droplet microfluidics technology is mainly used in droplet digital polymerase chain reaction (ddPCR) [10], enzyme analysis [11], and drug screening [12]. Some microfluidic devices for detecting bladder cancer cells have been reported [13,14]; however, there have been no reports about a single-cell microscopic observation device for detecting tumor cells using droplet microfluidic technology. The use of evidence-based medicines for bladder cancer diagnosis relies on finding tumor cells from a patient’s organ tissues and urine [15]. The development of a detection system that automatically recognizes urine tumor cells will facilitate the examination of urinary tumors. The microfluidic device proposed here can be used to directly identify different cells using different morphological features under an optical microscope, similar to the automatic urine sediment analyzer. We previously reported a method used to detect hematological tumor cells and pleural effusion tumor mass cells based on microfluidics technology [16,17]. Based on previous experiments, this study applied microfluidic chip technology to establish a system for detecting urinary tumor cells.

## 2. Materials and Methods

### 2.1. Microfluidic Chip Setup and Design

A silicon-based mask fabricated using a conventional photolithography process was used to fabricate a polydimethylsiloxane (PDMS)-based chip. The PDMS layer was manufactured as follows: a prepolymer mixed with a curing agent (Sylgard 184, Dow Corning, Midland, MI, USA) in a ratio of 10:1 was poured into a prefabricated mold and cured at 80 °C for 2 h. The cured PDMS film was then carefully peeled off and fluidic inlets and outlets were made using a 1.0 mm biopsy punch. This mold was bonded to the glass substrate by an oxygen plasma process (Diener electronic, Ebhausen, Germany).

Two channels were used to input the carrier oil (TargetingOne Technology (Beijing) Corporation, Beijing, China) and form an angle of 120°, and a single water phase input channel was located in the center of the two oil phase channels. The width of the oil phase input channel was 90 µm and the width of the water phase input channel was 80 µm. The computer-assisted design (CAD, Autodesk, San Rafael, CA, USA) designs are provided as Supplementary Data. To better experiment with droplet generation and cell encapsulation, we set the total flow of the two oil phase input channels to 4 mL/h and the total flow of the water phase input channel to 2 mL/h.

### 2.2. Cell Culture

The T-24 cell line, whose cell expression is stable, is considered to be one of the cell lines which most strongly reflects the biological characteristics of bladder tumor cells [18]. MeT-5A is a normal human mesothelium cell line and is often used as a normal cell line for comparison against tumor cells [19]. T-24 and MeT-5A (Cell Resource Center, Shanghai Institutes for Biological Sciences, Chinese Academy of Sciences) were cultured for 4 days to prepare a cell suspension. The cell suspension was identified by trypan blue staining (live cells were not stained and were maintained in a normal shiny form. Dead cells were blue, swollen, and dull), and the cells were counted.

T-24 was cultured in Roswell Park Memorial Institute Medium1640 (RPMI1640), without L-glutamine or phenol red (Beijing solarbio science & technology Co., Ltd., Beijing, China) but with 10% fetal bovine serum, 100 U/mL penicillin, and 100 g/mL streptomycin (Thermo Fisher Scientific Inc., Waltham, MA, USA) in a standard humidified incubator with an atmosphere containing 5% CO_2_ at 37 °C.

MeT-5A was cultured in dulbecco’s modified eagle medium (DMEM (H)) with 4.5 g/L glucose, L- glutamine, 10% fetal bovine serum, 100 U/mL penicillin, and 100 g/mL streptomycin and without sodium pyruvate (Beijing Solarbio Science & Technology Co., Ltd.) in a standard humidified incubator with an atmosphere containing 5% CO_2_ at 37 °C.

### 2.3. Trypan Blue Staining

The cell suspension was mixed with 0.4% trypan blue solution (Beijing Solarbio Science & Technology Co., Ltd.) at a ratio of 9:1. It was stained for 3 min, and 10 µL was pipetted and counted three times on a hemocytometer plate.

### 2.4. Image Characteristics of Cells

Matlab software (The MathWorks, Inc; Natic, MA, USA) was used to analyze the characteristic parameters of the cells, and the energy standard deviation and color mean were analyzed for the T-24 and MeT-5A cell images, respectively [20,21].

The gray-scale co-occurrence matrix shows the joint probability distribution of two gray-scale pixels at a distance d in the image. The conditional probability reflects the texture, which is the representation of the gray correlation of adjacent pixels. I is a two-dimensional digital image. Its size is M×N and the gray level is L. The gray level of each point in the image can be expressed as I(x,y). Two points are taken randomly in the image (k,l), (m,n), and form a point pair, setting the gray value of the pair as (i,j), I(k,l)=i, I(m,n)=j. Then, the gray level co-occurrence matrix P is a square matrix of L×L, and the elements in the square matrix are defined as

(1) P(i,j,d,θ)=#{((k,l),(m,n))∈Ω|I(k,l)=i,I(m,n)=j}

where d=max(|l−m|,|k−n|) is the maximum values of the horizontal and vertical coordinate distances of the two pixel points. θ is the angle between two pixels, which is usually selected from the east–west, northwest–southeast, south–north, and northeast–southwest directions (starting from the upper left corner), also known as 0°, 45°, 90°, and 135°.

The energy provides the sum of the square elements in the gray level co-occurrence matrix, which is a measure of the stability of the gray level change in the image texture, reflecting the uniformity of the image gray distribution and the texture thickness. A large energy value indicates that the current texture is a stable texture, also known as consistency.

(2) $$Ene=\overset{L-1}{\underset{i=0}{\sum }}\overset{L-1}{\underset{i=0}{\sum }}{\left(P\left(i,j\right)\right)}^{2}$$

where P(i,j) is the value of the *i*th row and the *j*th column element in the gray level co-occurrence matrix L represents the total number of gray levels in the image.

The color histogram is a common method used to characterize the color distribution and describe the proportions of different colors that occupy the entire image.

(3) $$H\left(i\right)=\frac{n_{i}}{N},i=0,1,\ldots ,L-1$$

(4) $$u=\overset{L-1}{\underset{i=0}{\sum }}iH\left(i\right)$$

where i represents the gray level to which the pixel belongs, L represents the total gray level, $n_{i}$ denotes the number of pixels having gray level i, and N represents the total number of pixels in the image.

### 2.5. Statistical Analysis

All data were processed using SPSS22.0 (IBM Corp. Armonk, NY, USA), and the results are expressed as the mean ± standard deviation (x±s). The results were compared using two independent samples *T*-tests, and p<0.05 was considered statistically significant.

## 3. Results

### 3.1. Device Setup and Working Principle

**Figure 1:**
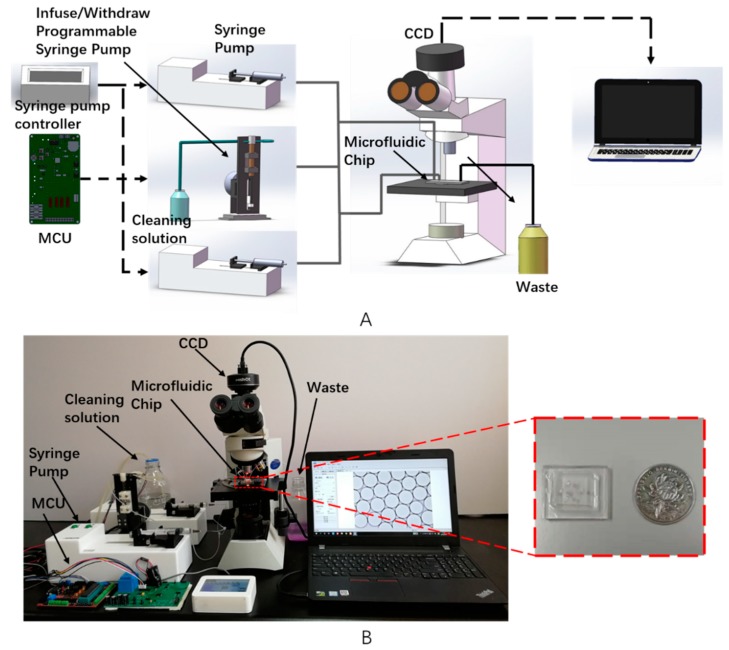
(**A**) Schematics and (**B**) a prototype of the microfluidic detection system for bladder cancer tumor cells, consisting of a single chip microcomputer, a syringe pump, a programmable infusion/withdrawal syringe pump, a microscope, a charge coupled device (CCD), and a computer. The inset shows a polydimethylsiloxane (PDMS) microfluidic chip.

As shown in Figure 1, the system consists of a microfluidic chip, a driving device, a microfluidic operation controller, a test reagent, a charge coupled device CCD (Guangzhou Mshot Photoelectric Technology CO., LTD., Guangzhou, China) cell morphology collector, an optical microscope, and a software system. The whole system registers the encapsulation of urine cells into the reaction reagent and classifies them according to the morphological characteristics of tumor cells, achieving the goal of screening and identifying bladder cancer tumor cells. The principle is that the reagent enters the microfluidic chip precisely and on time through a three-way channel. The droplets of the wrapped cells are generated in the chip and simultaneously captured by the CCD camera and finally analyzed on the computer. The workflow can be summarized as injecting the cleaning solution into the chip through a self-made syringe pump, pre-cleaning so that the cleaning solution is stopped, and then injecting the oil and the sample into the chip through a syringe pump (Suzhou Wenhao Microfluidic Technology co., Ltd., Suzhou, China) (Video S1). The CCD monitors the droplet generation process (Video S2) and observes the droplets at the exit, and the droplets can also be collected and observed outside the chip. After completing the test, injection of the oil and sample is stopped, and then the solution is cleaned again (Video S3).

### 3.2. Cell Concentration and Viability

**Figure 2:**
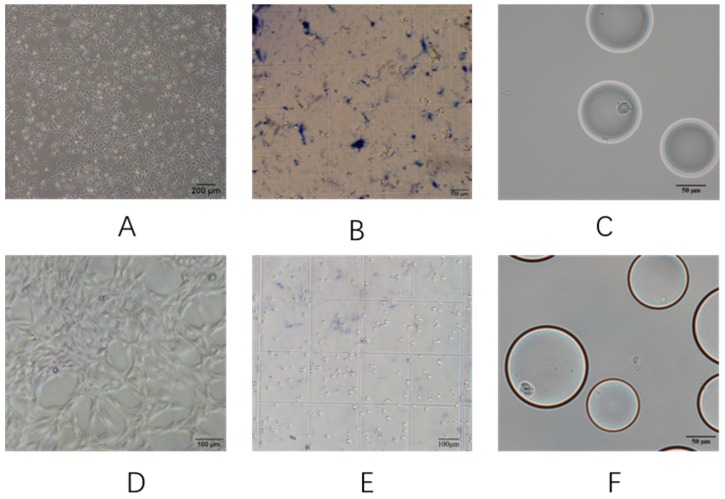
Images of the cell culture stained with trypan blue and packaged in droplets. (**A**–**C**) show images of T-24 cells; (**D**–**F**) show images of MeT-5A cells.

The cell suspension was identified by trypan blue staining, and the cells were counted. The concentration of the T-24 cell suspension was 837.5 cells/µL and the cell viability was 95.67 ± 1.25%, while the concentration of the MeT-5A cell suspension was 1650 cells/µL, and the cell viability was 95 ± 0.82%. Figure 2A,B,D,E shows the cell culture and trypan blue staining experimental images.

### 3.3. Analysis of Tumor Cells Based on Cell Image Characteristics

After 4 days of T24 and MeT-5A cell culture, the cultured cells were collected. The CCD images were obtained by a microfluidic detection system, as shown in Figure 2C,F, and a total of 100 cells were counted. The energy standard deviation and color mean of tumor cells and non-tumor cells were calculated using self-designed cell characterization software and were compared with independent *t*-tests. There was a significant difference in the energy standard deviation eigenvalues between the two cells, as shown in Table 1. The experimental results of the cellular characteristics in the urine analyzed by the urine microfluidic detection system suggest that the energy standard deviation of the cell image based on the urine cells can distinguish between tumor cells and non-tumor cells in the urine. In this way, based on microfluidic chip technology, the urine tumor is finally separated, and the characteristics of the cell biological expression substance are combined to realize the identification of the bladder cancer tumor cells.

### 3.4. Performance of Bladder Cancer Cell Detection

**Figure 3:**
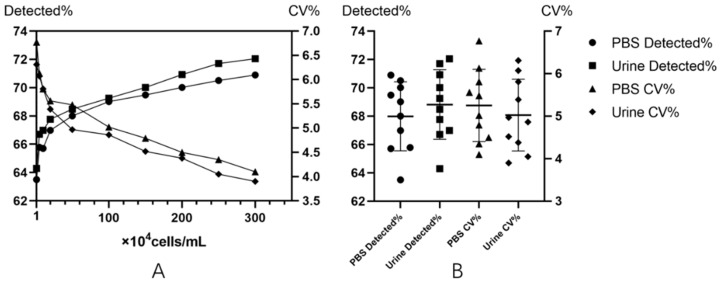
The detection and repeatability were superior in the urine samples compared with the PBS samples. Solid circles represent detected rate with T-24 cells only in the PBS and solid squares represent T-24 cells detected in the urine. Solid triangles represent CV% with T-24 cells only in the PBS and solid diamonds represent T-24 cells in the urine. (**A**) The left vertical axis shows the detected rate (i.e., number of cells detected versus number of cells spiked in) of T-24 cells. The right vertical axis shows the CV% (i.e., variation of in multiple parallel experiments) of detected rates. (**B**) Comparison of the detected rates and reproducibility (CV%) of T-24 cells in PBS samples and urine samples.

Phosphate buffer saline (PBS) at pH 7.4 is a suitable human body fluid that allows for survival in a physiological environment. The cell suspension prepared was used to simulate the working state of the system without interference from other substances. T24 cells were mixed in PBS and configured to have a total concentration of 1 × 10^4^ to 300 × 10^4^ per mL. Each time, 0.5 mL of the above-mentioned cells was tested, the test was repeated five times at each concentration, and the number of tumor cells per test was counted. Epidemiological evidence shows that 80% to 90% of patients with bladder cancer have hematuria [22]; therefore, in order to more realistically reflect the clinical situation, 0.5 mL of the above-mentioned concentration of T24 cells was concentrated to 0.1 mL and then added to the same 0.4 mL of a mixed hematuria sample containing leukocytes, squamous epithelium, crystals, etc., and each was tested five times. The average time taken for 100 tests was 15.02 ± 1.04 min. The detected rates and CV% of PBS and urine samples were tested by *t*-tests. There was no statistically significant difference between the two detected rates (*p* = 0.962). There was no significant difference in CV% (*p* = 0.984). This indicates that there was no statistically significant difference in the detection of this system regardless of the type of specimen in the cell or the interference of other cells in the specimen. The data are detailed in Table 2 and Figure 3.

**Figure 4:**
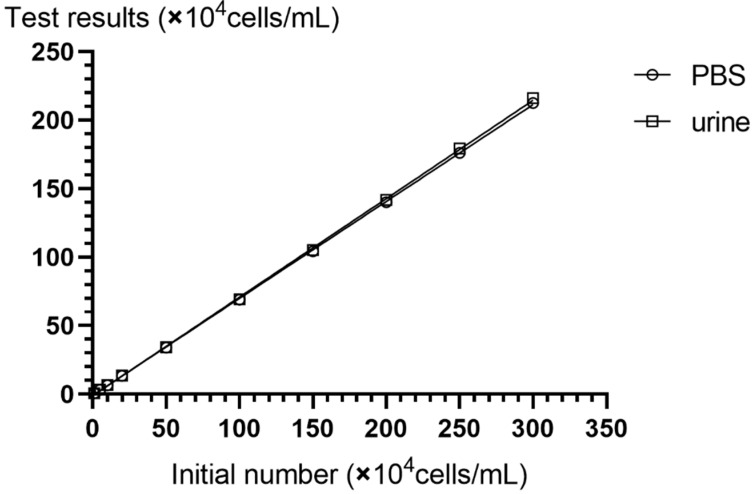
The abscissa indicates the number of cells initially added per milliliter, and the ordinate is the number of cells per milliliter of two different samples after detection. Both samples maintained good linearity with the sample to be tested.

The consistency between the detection results of the method and the specimens added was investigated, and the correlation between the number of cells before and after the detection of the two samples was analyzed, as shown in Figure 4. When the test sample was PBS, the correlation coefficient was *R*^2^ = 0.998, and the linear regression equation was Y = 0.705X − 0.098; when the sample was urine, the correlation coefficient was R^2^ = 0.999, and the linear regression equation was Y = 0.716X − 0.210.

## 4. Discussion

Through the advancement of bladder cancer diagnostic techniques, more and more biomarkers have been found [23,24]. However, urine cytology still contributes to disease diagnosis as a classic, inexpensive, and rapid detection method [25]. Here, the proposed system detected the sample at a speed of 2 mL/h, and according to CLSI Urinalysis approved guidelines, the detection time for a concentrated 1 mL sample was only 0.5 h [26]. As a non-invasive in vitro biopsy method, urine cytology takes significantly less time than the current histopathological examination method [27] and is less damaging to patients than imaging methods such as imaging and cystoscopy. The image recognition technology used in this paper can improve the low sensitivity of urine cytology caused by human factors to some extent [28]. The composition of human urine is rich and complex; in addition to cells, it contains components such as salt crystals and bacteria, which are obstacles to urine cell analysis. As a result, researchers have mitigated this negative impact using different methods [29,30]. The encapsulation of cells into droplets enables single-cell analysis and is easier to image than conventional sheath fluid. To observe the reliability of tumor analysis in this experiment, the correlation between the detected value and the original value was linearly correlated and regressed, and the results showed a good linear relationship. To explore the interference factors of tumor cells in this experiment, the effects of PBS and urine as two samples for cell detection were compared. The difference in detection and repeatability between the two sample types was not statistically significant. This is of great significance for subsequent clinical promotion. The detected rate of this design has yet to be improved, and some solutions have been reported by overwhelming the Poisson distribution of droplets into single-cell packages [31,32,33,34], which is beneficial to further improving the system’s performance.

## 5. Conclusions

We proposed a system to detect urinary tumor cells based on droplet microfluidic chips. By using the characteristics of the droplet chip single-cell analysis, the interference of other urinary substances is removed. The system can operate quickly, testing 2 mL specimens per hour. The technique based on microfluidic detection of urinary tumor cells proposed in this paper provides a new procedure for bladder cancer diagnosis.

## Figures and Tables

**Table 1:** Image characteristics of stained T-24 and MeT-5A cells.

Characteristic Parameters	Energy Variance (*n* = 100)	Color Mean (*n* = 100)
T-24	7.914 ± 1.665	7.067 ± 0.048
MeT-5A	4.624 ± 1.375	7.103 ± 0.036
t	21.54	0.2357
*p*	0.000	0.814

**Table 2:** Performance of bladder cancer cell detection in phosphate buffer saline (PBS) and urine.

T24 Cells (10^4^ per mL)	$\mathrm{Cells}\;(x\times 10^{4}\pm sd\times 10^{4})$		Detected%		CV%	
	PBS	Urine	PBS	Urine	PBS	Urine
1	0.64 ± 0.04	0.64 ± 0.04	63.50	64.30	6.77	6.31
5	3.28 ± 0.20	3.34 ± 0.20	65.78	66.71	6.13	6.07
10	6.58 ± 0.38	6.7 ± 0.38	65.71	66.99	5.81	5.81
20	13.4 ± 0.74	13.56 ± 0.74	66.98	67.77	5.56	5.39
50	34 ± 1.86	34.24 ± 1.70	68.00	68.49	5.48	4.97
100	69 ± 3.46	69.2 ± 3.36	69.01	69.25	5.02	4.86
150	104.24 ± 5.00	105.04 ± 4.74	69.49	70.02	4.79	4.52
200	140.02 ± 6.30	141.84 ± 6.22	70.01	70.92	4.50	4.38
250	176.24 ± 7.66	179.24 ± 7.26	70.50	71.70	4.35	4.05
300	212.68 ± 8.72	216.16 ± 8.44	70.89	72.05	4.10	3.90

## Supplementary Materials

The following are available online at https://www.mdpi.com/2072-666X/10/12/871/s1, Video S1: Sample injection, Video S2: Droplet generation, Video S3: Cleaning.
